# Health effects of the September 2009 dust storm in Sydney, Australia: did emergency department visits and hospital admissions increase?

**DOI:** 10.1186/1476-069X-12-32

**Published:** 2013-04-16

**Authors:** Alistair Merrifield, Suzanne Schindeler, Bin Jalaludin, Wayne Smith

**Affiliations:** 1 Centre for Epidemiology and Evidence, New South Wales Ministry of Health, Sydney, Australia; 2 Centre for Research, Evidence Management and Surveillance, Sydney South West Area Health Service, Sydney, Australia; 3 School of Public Health and Community Medicine, University of New South Wales, Sydney, Australia; 4 Environmental Health Branch, New South Wales Ministry of Health, Sydney, Australia

**Keywords:** Air pollution, Emergency department, Hospital admissions, Dust storm, Distributed-lag

## Abstract

**Background:**

During September 2009, a large dust storm was experienced in Sydney, New South Wales, Australia. Extremely high levels of particulate matter were recorded, with daily average levels of coarse matter (<10 μm) peaking over 11,000 μg/m^3^ and fine (<2.5 μm) over 1,600 μg/m^3^. We conducted an analysis to determine whether the dust storm was associated with increases in all-cause, cardiovascular, respiratory and asthma-related emergency department presentations and hospital admissions.

**Methods:**

We used distributed-lag Poisson generalized models to analyse the emergency department presentations and hospital admissions adjusted for pollutants, humidity, temperature and day of week and seasonal effects to obtain estimates of relative risks associated with the dust storm.

**Results:**

The dust storm period was associated with large increases in asthma emergency department visits (relative risk 1.23, 95% confidence interval 1.10-1.38, p < 0.01), and to a lesser extent, all emergency department visits (relative risk 1.04, 95% confidence interval 1.03-1.06, p < 0.01) and respiratory emergency department visits (relative risk 1.20, 95% confidence interval 1.15-1.26, p < 0.01). There was no significant increase in cardiovascular emergency department visits (p = 0.09) or hospital admissions for any reason. Age-specific analyses showed the dust storm was associated with increases in all-cause and respiratory emergency department visits in the ≥65 year age group; the ≤5 year group had higher risks of all-cause, respiratory and asthma-related emergency department presentations.

**Conclusions:**

We recommend public health measures, especially targeting asthmatics, should be implemented during future dust storm events.

## Background

A large growing body of literature supports the association between exposure to particulate air pollution and adverse health outcomes [1-10]. Reports discriminate, to some extent, between health outcomes associated with different sources of particulate matter (PM). Relatively few published reports concentrate on exposure to primarily coarse respirable particles (PM_10_), less than 10 μm in diameter [4,10-14]. These reports are generally associated with geological dust, in areas where dust exposure is frequently recurring and there are a large number of days of moderate to high exposure available to investigate potential health outcomes.

An issue with many studies is the potential mitigating effect of health warnings on high pollution days. Such warnings are designed to affect population behaviour to reduce the incidence of adverse health outcomes. Warnings are commonly disseminated during high PM air pollution episodes. On Wednesday 23rd September 2009, the city of Sydney (in New South Wales (NSW), Australia), experienced a rare large dust storm [15]. Extremely high levels of PM were recorded, with levels of PM_10_ and fine PM (less than 2.5 μm in diameter, PM_2.5_) peaking at over 11,000 μg/m^3^ and 1,600 μg/m^3^ (daily average levels), respectively. PM_10_ and PM_2.5_ levels experienced during this dust storm exceeded any extreme PM levels during bush fires and dust storms in the previous 15 years, by several orders of magnitude [7,8]. The dust storm dissipated considerably by early evening but two days later another less severe dust storm event lasting approximately 24 hours occurred. Exhaustive media coverage advised of health risks and mitigation options – avoid exposure if possible; stay indoors (in air conditioned buildings); do not exercise; follow asthma plans and seek medical help if respiratory or cardiovascular symptoms occurred. These messages were widely disseminated in mainstream media, and SMS text alerts were sent to those subscribing to the NSW Office of Environment and Heritage (OEH) air quality alert website [16].

Because of the sudden onset of extreme levels of PM, this dust storm provides an opportunity to assess morbidities during an extreme air pollution event that received widespread media attention. We conducted an analysis to determine associations between this dust storm and outcomes of emergency department (ED) presentations and hospital admissions for all causes and specific sub-groups of cardiovascular disease, respiratory disease and asthma.

## Methods

### Hospital and air pollution data

Data were restricted to the Sydney Statistical Division (SSD) from 1 January 2004 to 31 December 2009 (a total of 2191 days). The SSD is a standard geographical classification defined by the Australia Bureau of Statistics and contains the metropolitan area of Sydney (the largest city in Australia and the capital of NSW). In 2008, the SSD had an estimated population of 4,339,722 persons, consisting of 1,423,521 households with the majority of residents living in an urban environment (97.5%). 18.9% of the population were aged 14 and under and 12.1% aged 65 and over [17].

Daily data on ED presentations and hospitalisations were obtained from the NSW Ministry of Health (NSW Health) and included information from 29 public hospitals in the SSD (over 95% of ED presentations in this region, pers. comm. NSW Health). Planned or pre-arranged visits were excluded from the analyses. The data was classified into cardiovascular disease (ICD-9-CM: 036.42, 074.22, 093.2, 098.84, 112.81, 115.04, 115.14, 115.94, 390-459, 785, 786.5, 793.2, 794.3; ICD-10-AM: B37.6, G45, I00-I99, R00-R03, R07.1-R07.4, R93.1, R94.3, Z86.7), respiratory disease (ICD-9-CM: 033, 460-519, 768-770, 786.0-786.4, 799.1; ICD-10-AM: A37, J00-J99, P20-P28, R04.2-R06, R09.0-R09.3, R09.89) and asthma (ICD-9-CM: 493; ICD-10-AM: J45-J46) daily counts.

Daily air pollution data was obtained from NSW OEH on PM_10_ and PM_2.5_, carbon monoxide (CO), nitrogen dioxide (NO_2_), sulfur dioxide (SO_2_), ozone (O_3_), humidity and temperature from 19 air pollution monitoring stations located in the SSD. The pollutant data were summarised as daily means of data from monitoring stations during the study period. The pollutant and meteorological data from the recording stations provided by OEH were complete for the SSD area.

In this study we used routinely collected de-identified administrative hospitalisation and ED visit datasets and therefore we did not require approval from an institutional ethics committee. The NSW Ministry of Health, as the data custodian for these two datasets, approved the use of the datasets.

### Statistical analysis

Data was analysed using R 2.15.1 (The R Foundation for Statistical Computing) with the ‘dlnm’ package.

Descriptive statistics for air pollutants, humidity, temperature, ED presentations and hospital admissions for each of the 4 month periods during 1 August 2004 to 30 November 2009 were analysed to compare with corresponding levels on the day of the dust storm. August is the final month of winter and November is the end of spring in the southern hemisphere, these are the surrounding months of the dust storm. Outcomes were total number of daily ED presentations and daily hospital admissions for all-causes and for cardiovascular disease, respiratory disease and asthma.

An indicator variable from 23 September 2009 to 6 October 2009 (the dust storm period) was created to estimate associations between the dust storm and the ED presentations and hospital admissions. The high PM level on the day of the storm is problematic to model, the indicator variable is designed to capture the extreme PM levels occurring on the day of the storm and after-effects. CO, NO_2_, SO_2,_ O_3_, humidity and temperature levels were considered as potential confounders. These air pollution and meteorological variables were included as distributed lags, where multiple lags are simultaneously included in the time series model [18-20]. Distributed lag models have been used to previously model effect of air pollutants on health effects [21-24]. An indicator variable for the day of week was included to capture daily variation. To model seasonality and remove autocorrelation, we used a natural cubic spline smoothing function [25] for the date of the count. Degrees of freedom for the spline functions were selected via assessment of model fit using based on Akaike information criterion and residual autocorrelation.

The statistical analyses used distributed-lag Poisson Generalized Models [26] to model the count data. The maximum distributed-lag and degrees of freedom for the cubic spline were chosen by assessing model fit with modified Akaike and Bayesian information criteria [23]. Relative risks (RR), 95% confidence intervals (CI’s) and p-values (Wald Type III p-values) were calculated. The results presented in this article are from the final model derived from this variable selection process, listings of variables included in the model are reported in tables in Additional file 1.

We examined residual plots (quantile-quantile plots, histograms and plots of residuals against fitted and additive predictors) and goodness of fit statistics to check model diagnostics [26]. We also examined the effect of the dust storm period on two age groups (0-5 years, 65 years and older) and on sex. We performed sensitivity analyses on the model results by different smoothing methods and altering window length of the indicator for the dust storm period.

## Results

Cardiovascular disease ED presentations comprised 8.4%, respiratory 9.8% and asthma 1.2% of total ED presentations; cardiovascular disease admissions 9.9%, respiratory 12.9% and asthma 1.2% of total hospital admissions. Descriptive statistics for daily mean pollutant levels, temperature and humidity are presented in Table 1, showing comparisons of concentrations on the dust storm day (23/9/09) with levels in the dust storm period (23/9/09-6/10/09), 2009 and previous years (2004-2009). PM levels on the day of the dust storm were extremely high (daily average PM_10_ = 11 705 μg/m^3^; daily average PM_2.5_ = 1638 μg/m^3^). A second day of elevated PM levels occurred on 26/9/09 (daily average PM_10_ = 783 μg/m^3^; daily average PM_2.5_ = 110 μg/m^3^). The levels of PM during the dust storm were of an unprecedented order of magnitude higher than those experienced during previous years. Other pollutants, humidity and temperature levels were similar to levels in 2009 and previous years. There were no bushfire events, heat waves or other unusual metrological phenomena during the dust storm period [27]. Correlations between air pollutants, temperature and humidity are reported in Additional file 1: Table S1.

**Table 1:** Descriptive statistics of pollutant levels

**Pollutant**	**N**	**Min**	**Median**	**IQR**	**Mean**^**a**^	**Std Dev**	**Max**
**August**-**November 2004**-**2008**							
CO (ppm)	610	0.06	0.36	0.29	0.42	0.23	1.6
NO_2_ (ppbhm)	610	0.55	2.05	0.79	2.05	0.61	4.15
O_3_ 1 hourly (ppbhm)	610	1.57	3.38	1.08	3.62	0.95	8.73
PM_10_ (μg/m^3^)	610	10.57	34.69	18.53	38.84	23.13	258.18
PM_2.5_ (μg/m^3^)	610	3.3	13.83	9.47	16.08	12.39	174.42
SO_2_ (ppbhm)	610	0	0.21	0.21	0.25	0.17	0.9
Rel. humidity (%)	610	26.5	65.89	16.67	64.63	12.58	91.26
Temperature (C)	610	7.12	15.45	5.98	15.66	3.95	28.28
**August**-**November 2009**							
CO (ppm)	122	0.13	0.47	0.36	0.54	0.25	1.24
NO_2_ (ppbhm)	122	0.25	1.81	0.92	1.85	0.62	3.33
O_3_ 1 hourly (ppbhm)	122	2.03	3.25	0.73	3.42	0.73	5.99
PM_10_ (μg/m^3^)	122	13.95	38.48	31.42	165.75	1156.62	11704.8
PM_2.5_ (μg/m^3^)	122	4.22	14.66	15.53	34.13	161.05	1637.82
SO_2_ (ppbhm)	122	0	0.22	0.18	0.24	0.18	0.89
Rel. humidity (%)	122	35.29	65.97	18.4	63.88	14.16	89
Temperature (C)	122	8.37	15.37	5.05	15.41	3.41	27.5
**23**/**9**/**09**-**6**/**10**/**09**							
CO (ppm)	14	0.13	0.36	0.3	0.44	0.23	0.82
NO_2_ (ppbhm)	14	0.25	1.49	1.06	1.63	0.83	3.18
O_3_ 1 hourly (ppbhm)	14	2.7	3.11	0.34	3.2	0.41	4.12
PM_10_ (μg/m^3^)	14	14.07	47.57	43.75	925.25	3108.95	11704.8
PM_2.5_ (μg/m^3^)	14	5.58	12.5	18.3	137.85	432.57	1637.82
SO_2_ (ppbhm)	14	0	0.11	0.19	0.13	0.1	0.29
Rel. humidity (%)	14	35.58	52.2	38.12	59.51	20.64	88.54
Temperature (C)	14	12.2	14.65	3.27	15.19	2.46	20.8
**23**/**09**/**2009**^**b**^							
CO (ppm)	1				0.33		
NO_2_ (ppbhm)	1				0.56		
O_3_ 1 hourly (ppbhm)	1				2.98		
PM_10_ (μg/m^3^)	1				11704.8		
PM_2.5_ (μg/m^3^)	1				1637.82		
SO_2_ (ppbhm)	1				0.06		
Rel. humidity (%)	1				48		
Temperature (C)	1				17.52		

^a^Arithmetic mean. ^b^Consists of 1 measurement for the day 23/09/09.

The RRs for ED presentations from the modeling are shown in Table 2. The dust storm period is associated with a 4.3% increase in risk of all-cause ED presentations, compared to the remainder of the study period. The dust storm period was not significantly associated with cardiovascular ED presentations, but was associated with a 20% risk increase in respiratory ED presentations and a 23% increased risk increase of asthma ED presentations compared to other periods.

**Table 2:** Adjusted relative risks of ED presentations and hospital admissions associated with the 2009 dust storm

**ED Presentations**					**Hospital Admissions**				
**Category**	**RR**	**Lower 95% CI**	**Upper 95% CI**	**p**-**value**	**Category**	**RR**	**Lower 95% CI**	**Upper 95% CI**	**p**-**value**
**All**-**cause**					**All**-**cause**				
23/9/09-6/10/09	1.043	1.028	1.058	<.001	23/9/09-6/10/09	0.987	0.970	1.005	0.149
Non dust storm	1.000				Non dust storm	1.000			
**Cardiovascular**					**Cardiovascular**				
23/9/09-6/10/09	0.957	0.910	1.007	0.092	23/9/09-6/10/09	0.985	0.936	1.038	0.581
Non dust storm	1.000				Non dust storm	1.000			
**Respiratory**					**Respiratory**				
23/9/09-6/10/09	1.199	1.145	1.255	<.001	23/9/09-6/10/09	0.898	0.853	0.945	<.001
Non dust storm	1.000				Non dust storm	1.000			
**Asthma**					**Asthma**				
23/9/09-6/10/09	1.230	1.099	1.377	<.001	23/9/09-6/10/09	1.141	0.991	1.313	0.066
Non dust storm	1.000				Non dust storm	1.000			

Details of variables included and distributed-lags are included in Additional file 1: Tables S2-S9.

The corresponding RRs for hospital admissions from the modeling are shown in Table 2. The dust storm period was not associated with any significant increased risk of all-cause, cardiovascular or asthma hospital admissions. The dust storm period was associated with a 10% decrease in risk of respiratory hospital admissions, compared to other periods.

Results from the age-specific analyses for ED presentations are presented below. Amongst the ≥65 years group, the RR for all-cause ED presentations were increased compared to the results for all ages (RR 1.109, 95% CI 1.075-1.144, p < .001). There was no association between the dust storm and cardiovascular ED presentations (RR 0.940, 95% CI 0.872-1.013, p = 0.107). There were significant associations between the dust storm period and respiratory ED presentations (RR 1.281, 95% CI 1.174-1.397, p < .001) and none associated with asthma ED presentations in the ≥65 year group (RR 1.362, 95% CI 0.981-1.891, p = 0.065).

In the ≤5 year age group the RRs for all-cause ED presentations were increased compared to results from all ages and ≥65 year age group (RR 1.237, 95% CI 1.190-1.286, p < .001). RRs were not calculated for cardiovascular disease as there were too few events to calculate meaningful statistics. Compared to the ≥65 year age group, the ≤5 year age group had similar risk of respiratory (RR 1.286, 95% CI 1.192-1.392, p < .001) and asthma ED presentations (RR 1.268, 95% CI 1.041-1.545, p = 0.019).

Results from the sex-specific analyses for ED presentations are presented below. Increased risks were associated between the dust storm period and all-cause ED presentations for both sexes (compared to other periods), these effects are similar between the sexes (males: RR 1.095, 95% CI 1.074-1.116, p < .001; females: RR 1.144, 95% CI 1.121-1.168, p < .001). There were no significant associations between the dust storm period and cardiovascular ED presentations in either sex (males: RR 0.976, 95% CI 0.912-1.045, p = 0.489; females: RR 0.988, 95% CI 0.919-1.062, p = 0.749). The positive associations between the dust storm period and respiratory ED presentations are also similar between the sexes (males: RR 1.282, 95% CI 1.205-1.365, p < .001; females: RR 1.318, 95% CI 1.233-1.409, p < .001). Asthma hospital admissions for both sexes showed significant increased risks during the dust storm period (males: RR 1.254, 95% CI 1.066-1.475, p < .001; females: RR 1.418, 95% CI 1.227-1.638, p < .001), compared to other periods.

The results remained consistent regardless of smoothing methods used (splines, loess curves) for seasonal effects and distributed-lags, and to varying the length of the time window for the period of the dust storm.

## Discussion

We conducted an analysis to investigate whether a severe dust storm period was associated with increases in ED presentations and hospital admissions. After controlling for potential confounders, the dust storm period was associated with large significant increases in asthma ED presentations, and to a lesser extent, respiratory ED presentations, all-cause ED presentations. There were no significant increases in ED visits for cardiovascular disease. The dust storm period was not associated with an increase in asthma ED visits in the ≥65 year age group. It is possible that older people affected by asthma were more likely to have stayed indoors and provided with extra care. They were therefore less likely to be adversely affected by the dust storm. The dust storm was not associated with increases in risk for all-cause, cardiovascular or asthma hospital admissions. It is possible issues were not severe enough for subsequent admission to hospitals.

This dust storm originated in the Western NSW and South Australian arid zones and was clearly tracked over time during its travel path. There were no bush fire events or other unusual metrological phenomena during this period [27]. The study event is one very extreme event and it is difficult to find similar events to compare to. The Sydney region is prone to bushfires and these are best for comparison, although the levels of PM during these fires are 10 times lower. This dust storm appears to be the first well recorded event worldwide within a metropolitan area and therefore allows unique opportunities to study the health effects of the storm on the occupants of the metropolitan area [8].

Our results for all-cause, cardiovascular, and asthma hospital admissions are consistent with the current literature. Other studies have failed to find significant associations between dust storms and cardiovascular ED presentations and hospital admissions [28,29], which agree with our findings. We found increases in respiratory and asthma ED presentations during the dust storm period, which is in agreement with similar studies [9,30,31]. However our estimated effects were larger and we speculate these larger rates were due to the severity of the Sydney dust storm. Our study showed the dust storm period was associated with a decrease in respiratory hospital admissions. While the dust storm was associated with a higher risk of acute respiratory problems, these may not have been severe enough to warrant admission to hospital. Other studies examining respiratory admissions also did not find associations with dust storm events [10,28,32].

The majority of hospitals in the study area were included. Seasonality was controlled for by smoothing functions. We have no data on GP visits. The dust storm occurred on a Wednesday, GP clinics would have been open during this time. However, we are concerned with severe health symptoms and it is likely that GPs would have referred patients presenting with severe symptoms to an ED. Increased media awareness during the period of the dust storm may have biased results, warnings may have increased ED visits during this period due to over-cautiousness or decreased ED visits due to patients vacating the study area. We rely on the diagnoses coded in the NSW Health datasets, non-differential misclassification is unlikely due to the accuracy of records.

Public health messages were broadcast in the media in response to the dust storm to reduce the incidence of adverse health outcomes. Coverage included issuing warnings and recommendations to reduce the likelihood of adverse health outcomes: avoid exposure if possible, stay indoors (preferably in air conditioned buildings), do not exercise, follow asthma plans and seek medical help if respiratory or cardiovascular symptoms occurred. Because the dust storm and consequent public health messages had widespread media coverage, the health consequences from this dust storm are likely to represent the optimal health outcomes that can be hoped for when similar events occur in the future. Recent studies evaluating the health impact of media alerts on air quality among asthmatic patients [33] and the general population [34] suggest warnings may be associated with behavioural changes that reduce exposure to air pollutants. Our study is unable to evaluate the efficacy of these warnings; a study involving a survey soon after such an event would be the most appropriate way to evaluate the impact of media warnings [33]. Our study revealed that the dust storm period was associated with higher rates in asthma related ED visits. We recommend that specific warnings targeting asthmatics should be released prior to predicted future dust storm events.

We investigated the effects of the dust storm on the broad categories of respiratory and cardiovascular ED visits and hospital admissions. Future research could include determining the effect of the dust storm on specific conditions within these categories such as pneumonia, stroke, or myocardial infarctions. Furthermore, utilizing other data sources such as ambulance call-out data may complement our results obtained from ED and hospital data.

## Conclusions

The dust storm period was associated with large significant increases in asthma ED visits. Respiratory and all-cause ED visits also increased, although to a lesser extent. We recommend that public health measures including specific warnings targeting asthmatics should be implemented during future dust storm events.

## Abbreviations

CI: Confidence interval; ED: Emergency department; GP: General practitioner; ICD-9-CM: International classification of diseases, ninth revision, clinical modification; ICD-10-AM: International classification of diseases, tenth revision, Australian modification; NSW: New South Wales; NSW Health: NSW Ministry of Health; SSD: Sydney Statistical Division; OEH: Office of Environment and Heritage; PM: Particulate matter; PM2.5: Particulate matter with an aerodynamic diameter ≤ 2.5 μm; PM10: Particulate matter with an aerodynamic diameter ≤ 10 μm; RR: Relative risk.

## Competing interests

The authors declare that they have no competing interests.

## Authors’ contributions

All authors of this paper have critically read and approved the final version submitted. They have also made substantive intellectual contributions by directly participating either in the planning, execution, or analysis of the study. AM, BJ and WS contributed to the development of the study design, acquisition and interpretation of data and drafted the paper. AM did the statistical analysis and wrote the statistical analysis section of the paper. SS contributed substantially to acquisition and interpretation of data and was involved in drafting the manuscript. All authors have revised drafts and contributed to the revisions.

## Supplementary Material

Additional file 1: Table S1 Correlations between air pollutants, temperature and humidity. **Table S2.** Adjusted relative risks of all-cause ED presentations associated with the 2009 dust storm. Excludes reporting of smoothing cubic splines terms used for time. **Table S3.** Adjusted relative risks of cardiovascular ED presentations associated with the 2009 dust storm. Excludes reporting of smoothing cubic splines terms used for time. **Table S4.** Adjusted relative risks of respiratory ED presentations associated with the 2009 dust storm. Excludes reporting of smoothing cubic splines terms used for time. **Table S5.** Adjusted relative risks of asthma ED presentations associated with the 2009 dust storm. Excludes reporting of smoothing cubic splines terms used for time. **Table S6.** Adjusted relative risks of all-cause hospital admissions associated with the 2009 dust storm. Excludes reporting of smoothing cubic splines terms used for time. **Table S7.** Adjusted relative risks of cardiovascular hospital admissions associated with the 2009 dust storm. Excludes reporting of smoothing cubic splines terms used for time. **Table S8.** Adjusted relative risks of respiratory hospital admissions associated with the 2009 dust storm. Excludes reporting of smoothing cubic splines terms used for time. **Table S9.** Adjusted relative risks of asthma hospital admissions associated with the 2009 dust storm. Excludes reporting of smoothing cubic splines terms used for time. Click here for file

## References

[B1] Dockery D Pope C Acute respiratory effects of particulate air pollution Annu Rev Public Health 1994 15 107 132 10.1146/annurev.pu.15.050194.000543 8054077

[B2] Pope CA Dockery DW Schwartz J Review of epidemiological evidence of health effects of particulate air pollution Inhal Toxicol 1995 7 1 18 10.3109/08958379509014267

[B3] Jalaludin B Smith M O'Toole B Leeder SR Acute effects of bushfires on peak expiratory flow rates in children with wheeze: a time series analysis Aust N Z J Public Health 2000 24 174 177 10.1111/j.1467-842X.2000.tb00138.x 10790937

[B4] Yang CY Tsai SS Chang C-C Ho SC Effects of Asian dust storm events on daily admissions for Asthma in Taipei, Taiwan Inhal Toxicol 2005 17 817 821 10.1080/08958370500241254 16282159

[B5] Chen L Verrall K Tong S Air particulate pollution due to bushfires and respiratory hospital admissions in Brisbane, Australia Int J Environ Heal Res 2006 16 181 191 10.1080/09603120600641334 16611563

[B6] Morgan G Sheppeard V Khalaj B Ayyar A Lincoln D Jalaludin B Beard J Corbett S Lumley T Effects of Bushfire Smoke on Daily Mortality and Hospital Admissions in Sydney, Australia Epidemiology 2010 21 47 55 10.1097/EDE.0b013e3181c15d5a 19907335

[B7] Johnston FH Hanigan IC Henderson SB Morgan GG Portner T Williamson GJ Bowman MJS Creating an Integrated Historical Record of Extreme Particulate Air Pollution Events in Australian Cities from 1994 to 2007 J Air Waste Manag Assoc 2011 61 390 398 10.3155/1047-3289.61.4.390 21516934

[B8] Johnston FH Hanigan IC Henderson SB Morgan GG Bowman D Extreme air pollution events from bushfires and dust storms and their association with mortality in Sydney, Australia 1994–2007 Environ Res 2011 111 811 816 10.1016/j.envres.2011.05.007 21601845

[B9] Tam WWS Tze TW Wong AHS Hui DSC Effect of dust storm events on daily emergency admissions for respiratory diseases Respirology 2012 17 143 148 10.1111/j.1440-1843.2011.02056.x 22092966

[B10] Barnett AG Fraser JF Munck L The effects of the 2009 dust storm on emergency admissions to a hospital in Brisbane, Australia Int J Biometeorol 2012 56 719 726 10.1007/s00484-011-0473-y 21786212

[B11] Ostro BD Hurley S Lipsett MJ Air pollution and daily mortality in the Coachella Valley, California: a study of PM10 dominated by coarse particles Environ Res 1999 81 231 238 10.1006/enrs.1999.3978 10585019

[B12] Schwartz J Norris G Larson T Sheppard L Claiborne C Koenig J Episodes of high coarse particle concentrations are not associated with increased mortality Environ Health Perspect 1999 107 339 342 10.1289/ehp.99107339 10210688 PMC1566434

[B13] Kwon HJ Cho SH Chun Y Lagarde F Pershagen G Effects of the Asian dust events on daily mortality in Seoul Korea Environ Res 2002 90 1 5 10.1006/enrs.2002.4377 12359184

[B14] Chen YS Sheen PC Chenn ER Liu YK Wu TN Yang CY Effects of Asian dust storm events on daily mortality in Taipei Taiwan Environ Res 2004 95 151 155 10.1016/j.envres.2003.08.008 15147920

[B15] Radhi M Box MA Box GP Cohen DD Size-resolved chemical composition of the September 2009 Sydney dust storm Air Qual Climate Change 2010 44 25 30

[B16] NSW Office of Environment and Heritage Air quality[http://www.environment.nsw.gov.au/aqms/index.htm]

[B17] Australian Bureau of Statistics Sydney (Statistical Division) [http://www.abs.gov.au/ausstats/abs@.nsf/Previousproducts/105Population/People12004-2008?opendocument&tabname=Summary&prodno=105&issue=2004-2008&num=&view=]

[B18] Roberts S An investigation of distributed lag models in the context of air pollution and mortality time-series analysis J Air Waste Manage Assoc 2005 55 273 282 10.1080/10473289.2005.10464620 15828669

[B19] Zanobetti A Schwartz J Samoli E Gryparis A Touloumi G Peacock J The temporal pattern of respiratory and heart disease in response to air pollution Environ Health Perspect 2003 111 1188 1193 10.1289/ehp.5712 12842772 PMC1241573

[B20] Braga AL Zanobetti A Schwartz J The lag structure between particulate air pollution and respiratory and cardiovascular deaths in 10 U.S. cities Am J Repsir Crit Care Med 2001 179 1115 1120 10.1097/00043764-200111000-00001 11725331

[B21] Lall R Ito K Thurston GD Distributed lag analyses of daily hospital admissions and source-apportioned fine particulate air pollution Environ Health Perspect 2011 119 455 460 21172759 10.1289/ehp.1002638 PMC3080925

[B22] Goodman PG Dockery DW Clancy L Cause-specific mortaility and the extended effects of particulate pollution and temperature exposure Environ Health Perspect 2004 112 179 185 14754572 10.1289/ehp.6451 PMC1241827

[B23] Schwartz J The distributed lag between air pollution and daily deaths Epidemiology 2000 11 320 326 10.1097/00001648-200005000-00016 10784251

[B24] Wyzga RE The effect of air pollution upon mortality: a consideration of distributed lag models J Am Stat Assoc 1978 73 463 472 10.1080/01621459.1978.10480035 12262745

[B25] Hastie TJ Tibshirani RJ Generalized Additive Models 1990 Great Britain: Chapman & Hall

[B26] Gasparrini A Armstrong B Kenward MG Distributed lag non-linear models Stats Med 2010 29 2224 2234 10.1002/sim.3940 PMC2998707 20812303

[B27] Sydney Morning Herald Sydney turns red: dust storm blankets city 2009 [http://www.smh.com.au/environment/weather/sydney-turns-red-dust-storm-blankets-city-20090923-g0so.html]

[B28] Middleton N Yiallouros P Kleanthous S Kolokotroni O Schwartz J Dockery DW Demokritou P Koutrakis P A 10-year time-series analysis of respiratory and cardiovascular morbidity in Nicosia, Cyprus: the effect of short-term changes in air pollution and dust storms Environmental Health 2008 7 39 10.1186/1476-069X-7-39 18647382 PMC2517071

[B29] Yang CY Cheng MH Chen CC Effects of Asian dust storm events on hospital admissions for congestive heart failure in Taipei, Taiwan J Toxicol Environ Health Part A 2009 72 324 328 10.1080/15287390802529880 19184748

[B30] Bener A Abdulrazzaq YM Al-Mutawwa J Debuse P Genetic and environmental factors associated with asthma Hum Biol 1996 68 405 8935321

[B31] Delfino RJ Brummel S Wu J Stern H Ostro B Lipsett M Winer A Street DH Zhang L Tjoa T Gillen DL The relationship of respiratory and cardiovascular hospital admissions to the southern California wildfires of 2003 Occup Environ Med 2009 66 189 197 10.1136/oem.2008.041376 19017694 PMC4176821

[B32] Chiu HF Tiao MM Ho SC Kuo HW Wu TN Yang CY Effects of Asian dust storm events on hospital admissions for chronic obstructive pulmonary disease in Taipei, Taiwan Inhal Toxicol 2008 20 777 781 10.1080/08958370802005308 18645716

[B33] Wen XJ Balluz L Mokdad A Association between media alerts of air quality index and change of outdoor activity among adult asthma in six states, BFRSS 2005 J Community Health 2009 34 40 46 10.1007/s10900-008-9126-4 18821001

[B34] Kolbe A Gilchrist KL An extreme bushfire smoke pollution event: health impacts and public health challenges NSW Public Health Bulletin 2009 20 1–2 19 23 19261212 10.1071/nb08061

