# Supplementary material for: Health effects of the September 2009 dust storm in Sydney, Australia: did emergency department visits and hospital admissions increase?
Source: Environ Health. 2013 Apr 16;12:32. doi: 10.1186/1476-069X-12-32 (PMC3639126; doi:10.1186/1476-069X-12-32)
Supplement: Additional file 1: Table S1 — Correlations between air pollutants, temperature and humidity. Table S2. Adjusted relative risks of all-cause ED presentations associated with the 2009 dust storm. Excludes reporting of smoothing cubic splines terms used for time. Table S3. Adjusted relative risks of cardiovascular ED presentations associated with the 2009 dust storm. Excludes reporting of smoothing cubic splines terms used for time. Table S4. Adjusted relative risks of respiratory ED presentations associated with the 2009 dust storm. Excludes reporting of smoothing cubic splines terms used for time. Table S5. Adjusted relative risks of asthma ED presentations associated with the 2009 dust storm. Excludes reporting of smoothing cubic splines terms used for time. Table S6. Adjusted relative risks of all-cause hospital admissions associated with the 2009 dust storm. Excludes reporting of smoothing cubic splines terms used for time. Table S7. Adjusted relative risks of cardiovascular hospital admissions associated with the 2009 dust storm. Excludes reporting of smoothing cubic splines terms used for time. Table S8. Adjusted relative risks of respiratory hospital admissions associated with the 2009 dust storm. Excludes reporting of smoothing cubic splines terms used for time. Table S9. Adjusted relative risks of asthma hospital admissions associated with the 2009 dust storm. Excludes reporting of smoothing cubic splines terms used for time. [file 1476-069X-12-32-S1.docx]

**Table S1:** Correlations between air pollutants, temperature and humidity.

| **Pollutant** | **CO** | **NO_2_** | **O_3_** | **PM_10_** | **PM_2.5_** | **SO_2_** | **Temperature** | **Humidity** |
| --- | --- | --- | --- | --- | --- | --- | --- | --- |
| **CO (ppm)** |  | 0.613 | 0.031 | -0.009 | 0.060 | 0.382 | -0.223 | 0.008 |
| **NO_2_ (ppbhm)** | 0.613 |  | 0.400 | -0.077 | -0.010 | 0.530 | 0.019 | -0.179 |
| **O_3_ (ppbhm)** | 0.031 | 0.400 |  | 0.003 | 0.044 | 0.387 | 0.622 | -0.322 |
| **PM_10_ (µg/m³)** | -0.009 | -0.077 | 0.003 |  | 0.984 | -0.029 | 0.037 | -0.074 |
| **PM_2.5_ (µg/m³)** | 0.060 | -0.010 | 0.044 | 0.984 |  | 0.031 | 0.054 | -0.082 |
| **SO_2_ (ppbhm)** | 0.382 | 0.530 | 0.387 | -0.029 | 0.031 |  | 0.199 | -0.018 |
| **Temperature (C)** | -0.223 | 0.019 | 0.622 | 0.037 | 0.054 | 0.199 |  | -0.197 |
| **Rel. humidity (%)** | 0.008 | -0.179 | -0.322 | -0.074 | -0.082 | -0.018 | -0.197 |  |

**Table S2.** Adjusted relative risks of all-cause ED presentations associated with the 2009 dust storm. Excludes reporting of smoothing cubic splines terms used for time.

| **Variable** | **Category** | **RR** | **95% LCI** | **95% UCI** | **p-value** |
| --- | --- | --- | --- | --- | --- |
| 23/9/09-6/10/09 | | 1.043 | 1.028 | 1.058 | <.001 |
| Temperature | Lag 1 | 0.992 | 0.988 | 0.995 | <.001 |
|  | Lag 2 | 0.989 | 0.983 | 0.994 | <.001 |
|  | Lag 3 | 0.992 | 0.988 | 0.997 | 0.002 |
|  | Lag 4 | 0.995 | 0.990 | 1.000 | 0.037 |
|  | Lag 5 | 1.003 | 0.998 | 1.009 | 0.264 |
|  | Lag 6 | 0.987 | 0.977 | 0.998 | 0.018 |
|  | Lag 7 | 1.004 | 0.997 | 1.012 | 0.255 |
| Humidity | Lag 1 | 0.992 | 0.987 | 0.996 | <.001 |
|  | Lag 2 | 0.992 | 0.987 | 0.997 | <.001 |
|  | Lag 3 | 0.999 | 0.995 | 1.002 | 0.540 |
|  | Lag 4 | 0.983 | 0.972 | 0.994 | 0.002 |
|  | Lag 5 | 0.988 | 0.985 | 0.992 | <.001 |
| SO2 | Lag 1 | 0.999 | 0.995 | 1.003 | 0.521 |
|  | Lag 2 | 0.998 | 0.992 | 1.003 | 0.378 |
|  | Lag 3 | 0.994 | 0.989 | 0.998 | 0.004 |
|  | Lag 4 | 0.994 | 0.989 | 0.999 | 0.013 |
|  | Lag 5 | 1.006 | 1.001 | 1.010 | 0.020 |
|  | Lag 6 | 0.983 | 0.974 | 0.992 | <.001 |
|  | Lag 7 | 1.002 | 0.996 | 1.007 | 0.512 |
| O3 | Lag 1 | 0.992 | 0.983 | 1.001 | 0.082 |
|  | Lag 2 | 0.978 | 0.968 | 0.989 | <.001 |
|  | Lag 3 | 0.989 | 0.979 | 0.999 | 0.028 |
|  | Lag 4 | 0.980 | 0.969 | 0.990 | <.001 |
|  | Lag 5 | 0.988 | 0.982 | 0.994 | <.001 |
|  | Lag 6 | 0.945 | 0.923 | 0.966 | <.001 |
|  | Lag 7 | 0.992 | 0.982 | 1.002 | 0.113 |
| NO2 | Lag 1 | 1.057 | 1.047 | 1.066 | <.001 |
|  | Lag 2 | 1.064 | 1.054 | 1.074 | <.001 |
|  | Lag 3 | 1.058 | 1.047 | 1.068 | <.001 |
|  | Lag 4 | 1.034 | 1.027 | 1.041 | <.001 |
|  | Lag 5 | 1.138 | 1.115 | 1.162 | <.001 |
|  | Lag 6 | 1.017 | 1.005 | 1.029 | 0.006 |
| CO | Lag 1 | 0.998 | 0.993 | 1.003 | 0.493 |
|  | Lag 2 | 0.999 | 0.992 | 1.006 | 0.761 |
|  | Lag 3 | 0.996 | 0.990 | 1.002 | 0.234 |
|  | Lag 4 | 1.008 | 1.002 | 1.015 | 0.010 |
|  | Lag 5 | 1.001 | 0.995 | 1.007 | 0.674 |
|  | Lag 6 | 0.987 | 0.973 | 1.001 | 0.064 |
|  | Lag 7 | 1.000 | 0.992 | 1.008 | 0.975 |
| Day of week | Sunday | 1.073 | 1.067 | 1.078 | <.001 |
|  | Monday | 1.038 | 1.032 | 1.043 |  |
|  | Tuesday | 0.948 | 0.943 | 0.953 |  |
|  | Wednesday | 0.938 | 0.933 | 0.943 |  |
|  | Thursday | 0.935 | 0.930 | 0.940 |  |
|  | Friday | 0.959 | 0.954 | 0.964 |  |

**Table S3.** Adjusted relative risks of cardiovascular ED presentations associated with the 2009 dust storm. Excludes reporting of smoothing cubic splines terms used for time.

| **Variable** | **Category** | **RR** | **95% LCI** | **95% UCI** | **p-value** |
| --- | --- | --- | --- | --- | --- |
| Dust storm period indicator | | 0.957 | 0.910 | 1.007 | 0.092 |
| Temperature | Lag 1 | 1.008 | 0.995 | 1.020 | 0.231 |
|  | Lag 2 | 0.965 | 0.947 | 0.983 | 0.000 |
|  | Lag 3 | 0.979 | 0.963 | 0.996 | 0.013 |
|  | Lag 4 | 0.975 | 0.958 | 0.992 | 0.003 |
|  | Lag 5 | 0.987 | 0.969 | 1.007 | 0.196 |
|  | Lag 6 | 0.933 | 0.901 | 0.967 | 0.000 |
|  | Lag 7 | 0.977 | 0.952 | 1.002 | 0.068 |
| Humidity | Lag 1 | 0.954 | 0.939 | 0.969 | 0.000 |
|  | Lag 2 | 0.995 | 0.976 | 1.015 | 0.619 |
|  | Lag 3 | 0.970 | 0.952 | 0.989 | 0.002 |
|  | Lag 4 | 0.973 | 0.954 | 0.992 | 0.005 |
|  | Lag 5 | 0.986 | 0.972 | 1.001 | 0.070 |
|  | Lag 6 | 0.944 | 0.905 | 0.986 | 0.009 |
|  | Lag 7 | 0.972 | 0.958 | 0.985 | 0.000 |
| SO2 | Lag 1 | 0.944 | 0.905 | 0.986 | 0.397 |
|  | Lag 2 | 0.972 | 0.958 | 0.985 | 0.008 |
|  | Lag 3 | 0.995 | 0.984 | 1.007 | 0.007 |
|  | Lag 4 | 1.020 | 1.005 | 1.035 | 0.089 |
|  | Lag 5 | 0.983 | 0.970 | 0.995 | 0.330 |
|  | Lag 6 | 1.013 | 0.998 | 1.029 | 0.633 |
| O3 | Lag 1 | 1.013 | 0.987 | 1.040 | 0.006 |
|  | Lag 2 | 1.004 | 0.987 | 1.022 | 0.000 |
|  | Lag 3 | 0.957 | 0.928 | 0.987 | 0.012 |
|  | Lag 4 | 0.917 | 0.884 | 0.952 | 0.000 |
|  | Lag 5 | 0.957 | 0.925 | 0.990 | 0.002 |
|  | Lag 6 | 0.933 | 0.901 | 0.966 | 0.001 |
|  | Lag 7 | 1.034 | 1.012 | 1.056 | 0.785 |
| NO2 | Lag 1 | 0.873 | 0.807 | 0.944 | 0.920 |
|  | Lag 2 | 0.995 | 0.962 | 1.030 | 0.012 |
|  | Lag 3 | 1.002 | 0.970 | 1.035 | 0.465 |
|  | Lag 4 | 1.048 | 1.010 | 1.086 | 0.262 |
|  | Lag 5 | 1.013 | 0.979 | 1.047 | 0.402 |
|  | Lag 6 | 1.021 | 0.985 | 1.059 | 0.134 |
|  | Lag 7 | 1.010 | 0.987 | 1.034 | 0.000 |
| CO | Lag 1 | 1.059 | 0.982 | 1.142 | 0.257 |
|  | Lag 2 | 0.892 | 0.858 | 0.928 | 0.006 |
|  | Lag 3 | 1.011 | 0.992 | 1.029 | 0.000 |
|  | Lag 4 | 1.034 | 1.010 | 1.059 | 0.161 |
|  | Lag 5 | 1.040 | 1.018 | 1.062 | 0.000 |
|  | Lag 6 | 1.015 | 0.994 | 1.038 | 0.064 |
|  | Lag 7 | 1.068 | 1.049 | 1.087 | 0.000 |
| Day of week | Sunday | 1.046 | 0.997 | 1.097 | <.001 |
|  | Monday | 0.956 | 0.933 | 0.980 |  |
|  | Tuesday | 1.004 | 0.985 | 1.024 |  |
|  | Wednesday | 1.268 | 1.245 | 1.292 |  |
|  | Thursday | 1.185 | 1.163 | 1.208 |  |
|  | Friday | 1.175 | 1.153 | 1.197 |  |

**Table S4.** Adjusted relative risks of respiratory ED presentations associated with the 2009 dust storm. Excludes reporting of smoothing cubic splines terms used for time.

| **Variable** | **Category** | **RR** | **95% LCI** | **95% UCI** | **p-value** |
| --- | --- | --- | --- | --- | --- |
| 23/9/09-6/10/09 | | 1.199 | 1.145 | 1.255 | <.001 |
| Temperature | Lag 1 | 0.980 | 0.969 | 0.991 | <.001 |
|  | Lag 2 | 0.994 | 0.976 | 1.012 | 0.503 |
|  | Lag 3 | 0.980 | 0.965 | 0.995 | 0.008 |
|  | Lag 4 | 0.993 | 0.977 | 1.009 | 0.375 |
|  | Lag 5 | 0.958 | 0.940 | 0.976 | <.001 |
|  | Lag 6 | 1.004 | 0.972 | 1.037 | 0.811 |
|  | Lag 7 | 0.973 | 0.949 | 0.998 | 0.036 |
| Humidity | Lag 1 | 0.971 | 0.958 | 0.985 | <.001 |
|  | Lag 2 | 0.989 | 0.971 | 1.008 | 0.249 |
|  | Lag 3 | 0.972 | 0.957 | 0.988 | 0.001 |
|  | Lag 4 | 1.009 | 0.996 | 1.023 | 0.172 |
|  | Lag 5 | 0.981 | 0.943 | 1.020 | 0.326 |
|  | Lag 6 | 0.954 | 0.941 | 0.967 | <.001 |
| SO2 | Lag 1 | 0.988 | 0.976 | 1.000 | 0.057 |
|  | Lag 2 | 0.954 | 0.941 | 0.967 | 0.716 |
|  | Lag 3 | 0.988 | 0.976 | 1.000 | <.001 |
|  | Lag 4 | 1.003 | 0.986 | 1.021 | <.001 |
|  | Lag 5 | 0.968 | 0.954 | 0.982 | 0.005 |
|  | Lag 6 | 1.037 | 1.021 | 1.052 | 0.023 |
|  | Lag 7 | 0.978 | 0.963 | 0.993 | 0.325 |
| O3 | Lag 1 | 0.967 | 0.939 | 0.995 | 0.004 |
|  | Lag 2 | 1.009 | 0.991 | 1.027 | <.001 |
|  | Lag 3 | 0.959 | 0.931 | 0.987 | <.001 |
|  | Lag 4 | 0.940 | 0.908 | 0.972 | <.001 |
|  | Lag 5 | 0.931 | 0.902 | 0.961 | <.001 |
|  | Lag 6 | 0.937 | 0.907 | 0.968 | <.001 |
|  | Lag 7 | 0.907 | 0.888 | 0.926 | 0.267 |
| NO2 | Lag 1 | 0.802 | 0.746 | 0.861 | <.001 |
|  | Lag 2 | 0.982 | 0.950 | 1.014 | <.001 |
|  | Lag 3 | 1.161 | 1.125 | 1.197 | <.001 |
|  | Lag 4 | 1.174 | 1.135 | 1.215 | <.001 |
|  | Lag 5 | 1.188 | 1.150 | 1.227 | <.001 |
|  | Lag 6 | 1.146 | 1.107 | 1.187 | <.001 |
|  | Lag 7 | 1.111 | 1.087 | 1.137 | <.001 |
| CO | Lag 1 | 1.450 | 1.350 | 1.557 | <.001 |
|  | Lag 2 | 1.086 | 1.045 | 1.129 | <.001 |
|  | Lag 3 | 1.048 | 1.029 | 1.067 | <.001 |
|  | Lag 4 | 1.104 | 1.078 | 1.131 | <.001 |
|  | Lag 5 | 1.061 | 1.039 | 1.085 | <.001 |
|  | Lag 6 | 1.101 | 1.078 | 1.125 | <.001 |
|  | Lag 7 | 1.060 | 1.040 | 1.080 | 0.019 |
| Day of week | Sunday | 1.128 | 1.076 | 1.182 | <.001 |
|  | Monday | 0.972 | 0.949 | 0.995 |  |
|  | Tuesday | 1.160 | 1.141 | 1.179 |  |
|  | Wednesday | 1.119 | 1.100 | 1.138 |  |
|  | Thursday | 1.016 | 0.999 | 1.034 |  |
|  | Friday | 1.008 | 0.991 | 1.026 |  |

**Table S5.** Adjusted relative risks of asthma ED presentations associated with the 2009 dust storm. Excludes reporting of smoothing cubic splines terms used for time.

| **Variable** | **Category** | **RR** | **95% LCI** | **95% UCI** | **p-value** |
| --- | --- | --- | --- | --- | --- |
| 23/9/09-6/10/09 | | 1.230 | 1.099 | 1.377 | <.001 |
| Temperature | Lag 1 | 0.992 | 0.961 | 1.025 | 0.637 |
|  | Lag 2 | 1.048 | 1.000 | 1.098 | 0.050 |
|  | Lag 3 | 1.036 | 0.997 | 1.076 | 0.073 |
|  | Lag 4 | 1.002 | 0.958 | 1.048 | 0.932 |
|  | Lag 5 | 1.098 | 1.006 | 1.199 | 0.036 |
|  | Lag 6 | 1.032 | 0.969 | 1.100 | 0.323 |
| Humidity | Lag 1 | 0.957 | 0.923 | 0.993 | 0.019 |
|  | Lag 2 | 0.933 | 0.893 | 0.976 | 0.002 |
|  | Lag 3 | 0.971 | 0.941 | 1.002 | 0.068 |
|  | Lag 4 | 0.843 | 0.766 | 0.927 | <.001 |
|  | Lag 5 | 0.985 | 0.955 | 1.017 | 0.367 |
| SO2 | Lag 1 | 0.979 | 0.949 | 1.010 | 0.177 |
|  | Lag 2 | 1.024 | 0.987 | 1.063 | 0.209 |
|  | Lag 3 | 1.024 | 0.988 | 1.061 | 0.194 |
|  | Lag 4 | 1.038 | 0.971 | 1.109 | 0.269 |
|  | Lag 5 | 1.026 | 0.980 | 1.073 | 0.272 |
| O3 | Lag 1 | 0.862 | 0.797 | 0.933 | <.001 |
|  | Lag 2 | 0.877 | 0.798 | 0.965 | 0.007 |
|  | Lag 3 | 0.828 | 0.759 | 0.904 | <.001 |
|  | Lag 4 | 0.858 | 0.784 | 0.939 | 0.001 |
|  | Lag 5 | 0.855 | 0.809 | 0.903 | <.001 |
|  | Lag 6 | 0.699 | 0.573 | 0.852 | <.001 |
|  | Lag 7 | 1.008 | 0.925 | 1.100 | 0.850 |
| NO2 | Lag 1 | 0.989 | 0.927 | 1.055 | 0.732 |
|  | Lag 2 | 0.949 | 0.898 | 1.002 | 0.059 |
|  | Lag 3 | 0.928 | 0.800 | 1.076 | 0.323 |
|  | Lag 4 | 0.874 | 0.808 | 0.946 | 0.001 |
| CO | Lag 1 | 1.019 | 0.972 | 1.068 | 0.441 |
|  | Lag 2 | 1.123 | 1.054 | 1.197 | <.001 |
|  | Lag 3 | 1.100 | 1.039 | 1.164 | 0.001 |
|  | Lag 4 | 1.119 | 1.057 | 1.184 | <.001 |
|  | Lag 5 | 1.102 | 1.048 | 1.158 | <.001 |
|  | Lag 6 | 1.163 | 1.024 | 1.320 | 0.020 |
|  | Lag 7 | 0.984 | 0.923 | 1.049 | 0.622 |
| Day of week | Sunday | 1.245 | 1.188 | 1.304 | <.001 |
|  | Monday | 1.248 | 1.191 | 1.308 | <.001 |
|  | Tuesday | 1.094 | 1.043 | 1.148 | <.001 |
|  | Wednesday | 1.046 | 0.996 | 1.098 | 0.069 |
|  | Thursday | 0.985 | 0.937 | 1.035 | 0.548 |
|  | Friday | 0.960 | 0.913 | 1.009 | 0.108 |

**Table S6.** Adjusted relative risks of all-cause hospital admissions associated with the 2009 dust storm. Excludes reporting of smoothing cubic splines terms used for time.

| **Variable** | **Category** | **RR** | **95% LCI** | **95% UCI** | **p-value** |
| --- | --- | --- | --- | --- | --- |
| 23/9/09-6/10/09 | | 0.987 | 0.970 | 1.005 | 0.149 |
| Temperature | Lag 1 | 0.996 | 0.992 | 1.000 | 0.072 |
|  | Lag 2 | 0.992 | 0.986 | 0.998 | 0.011 |
|  | Lag 3 | 0.996 | 0.990 | 1.001 | 0.088 |
|  | Lag 4 | 0.994 | 0.988 | 1.000 | 0.037 |
|  | Lag 5 | 0.986 | 0.975 | 0.998 | 0.020 |
|  | Lag 6 | 0.990 | 0.981 | 0.998 | 0.016 |
| Humidity | Lag 1 | 0.998 | 0.993 | 1.004 | 0.534 |
|  | Lag 2 | 0.992 | 0.985 | 0.999 | 0.032 |
|  | Lag 3 | 1.004 | 0.998 | 1.011 | 0.192 |
|  | Lag 4 | 1.000 | 0.993 | 1.006 | 0.901 |
|  | Lag 5 | 1.007 | 1.002 | 1.012 | 0.012 |
|  | Lag 6 | 1.000 | 0.985 | 1.015 | 0.982 |
|  | Lag 7 | 0.981 | 0.976 | 0.986 | <.001 |
| SO2 | Lag 1 | 0.988 | 0.984 | 0.992 | <.001 |
|  | Lag 2 | 0.992 | 0.987 | 0.997 | 0.002 |
|  | Lag 3 | 0.978 | 0.973 | 0.983 | <.001 |
|  | Lag 4 | 1.003 | 0.997 | 1.008 | 0.309 |
|  | Lag 5 | 0.974 | 0.964 | 0.983 | <.001 |
|  | Lag 6 | 0.983 | 0.977 | 0.989 | <.001 |
| O3 | Lag 1 | 1.005 | 0.994 | 1.016 | 0.375 |
|  | Lag 2 | 0.991 | 0.979 | 1.004 | 0.182 |
|  | Lag 3 | 1.007 | 0.995 | 1.019 | 0.242 |
|  | Lag 4 | 1.003 | 0.991 | 1.015 | 0.668 |
|  | Lag 5 | 1.013 | 1.006 | 1.021 | 0.001 |
|  | Lag 6 | 0.990 | 0.964 | 1.017 | 0.461 |
|  | Lag 7 | 0.993 | 0.982 | 1.005 | 0.242 |
| NO2 | Lag 1 | 0.985 | 0.974 | 0.997 | 0.012 |
|  | Lag 2 | 0.978 | 0.966 | 0.991 | 0.001 |
|  | Lag 3 | 0.991 | 0.980 | 1.003 | 0.155 |
|  | Lag 4 | 0.984 | 0.972 | 0.997 | 0.013 |
|  | Lag 5 | 0.998 | 0.990 | 1.007 | 0.689 |
|  | Lag 6 | 0.976 | 0.951 | 1.001 | 0.062 |
|  | Lag 7 | 0.994 | 0.981 | 1.008 | 0.419 |
| CO | Lag 1 | 1.003 | 0.998 | 1.007 | 0.274 |
|  | Lag 2 | 1.006 | 1.000 | 1.012 | 0.037 |
|  | Lag 3 | 1.014 | 1.008 | 1.020 | 0.000 |
|  | Lag 4 | 1.010 | 0.998 | 1.022 | 0.108 |
|  | Lag 5 | 0.996 | 0.987 | 1.005 | 0.352 |
| Day of week | Sunday | 0.819 | 0.813 | 0.825 | <.001 |
|  | Monday | 1.491 | 1.481 | 1.501 |  |
|  | Tuesday | 1.493 | 1.483 | 1.503 |  |
|  | Wednesday | 1.488 | 1.478 | 1.498 |  |
|  | Thursday | 1.467 | 1.457 | 1.477 |  |
|  | Friday | 1.652 | 1.642 | 1.663 |  |

**Table S7.** Adjusted relative risks of cardiovascular hospital admissions associated with the 2009 dust storm. Excludes reporting of smoothing cubic splines terms used for time.

| **Variable** | **Category** | **RR** | **95% LCI** | **95% UCI** | **p-value** |
| --- | --- | --- | --- | --- | --- |
| 23/9/09-6/10/09 | | 0.985 | 0.936 | 1.038 | 0.581 |
| Temperature | Lag 1 | 0.984 | 0.972 | 0.996 | 0.011 |
|  | Lag 2 | 1.000 | 0.985 | 1.016 | 0.960 |
|  | Lag 3 | 0.971 | 0.957 | 0.985 | 0.000 |
|  | Lag 4 | 0.992 | 0.961 | 1.023 | 0.597 |
|  | Lag 5 | 0.966 | 0.940 | 0.992 | 0.010 |
| Humidity | Lag 1 | 0.991 | 0.975 | 1.006 | 0.242 |
|  | Lag 2 | 0.995 | 0.974 | 1.016 | 0.633 |
|  | Lag 3 | 1.008 | 0.989 | 1.027 | 0.424 |
|  | Lag 4 | 1.002 | 0.986 | 1.018 | 0.810 |
|  | Lag 5 | 1.027 | 0.983 | 1.074 | 0.230 |
|  | Lag 6 | 0.964 | 0.950 | 0.979 | <.001 |
| SO2 | Lag 1 | 0.982 | 0.967 | 0.996 | 0.012 |
|  | Lag 2 | 0.962 | 0.943 | 0.982 | <.001 |
|  | Lag 3 | 0.954 | 0.938 | 0.970 | <.001 |
|  | Lag 4 | 1.005 | 0.988 | 1.022 | 0.587 |
|  | Lag 5 | 0.950 | 0.933 | 0.967 | <.001 |
|  | Lag 6 | 0.918 | 0.887 | 0.950 | <.001 |
|  | Lag 7 | 1.002 | 0.981 | 1.022 | <.001 |
| O3 | Lag 1 | 0.964 | 0.932 | 0.996 | <.001 |
|  | Lag 2 | 0.920 | 0.884 | 0.957 | <.001 |
|  | Lag 3 | 0.947 | 0.913 | 0.982 | 0.003 |
|  | Lag 4 | 0.939 | 0.904 | 0.975 | 0.001 |
|  | Lag 5 | 0.932 | 0.911 | 0.955 | <.001 |
|  | Lag 6 | 0.809 | 0.745 | 0.879 | <.001 |
|  | Lag 7 | 0.993 | 0.957 | 1.030 | 0.703 |
| NO2 | Lag 1 | 1.008 | 0.979 | 1.037 | 0.606 |
|  | Lag 2 | 1.000 | 0.976 | 1.024 | 0.972 |
|  | Lag 3 | 1.028 | 0.961 | 1.100 | 0.422 |
|  | Lag 4 | 1.086 | 1.050 | 1.124 | <.001 |
| CO | Lag 1 | 1.046 | 1.024 | 1.067 | <.001 |
|  | Lag 2 | 1.069 | 1.040 | 1.098 | <.001 |
|  | Lag 3 | 1.049 | 1.023 | 1.076 | <.001 |
|  | Lag 4 | 1.069 | 1.044 | 1.096 | <.001 |
|  | Lag 5 | 1.051 | 1.029 | 1.075 | <.001 |
|  | Lag 6 | 1.105 | 1.046 | 1.167 | <.001 |
|  | Lag 7 | 0.998 | 0.972 | 1.024 | 0.857 |
| Day of week | Sunday | 0.826 | 0.806 | 0.847 | <.001 |
|  | Monday | 1.569 | 1.536 | 1.602 |  |
|  | Tuesday | 1.566 | 1.533 | 1.599 |  |
|  | Wednesday | 1.553 | 1.521 | 1.587 |  |
|  | Thursday | 1.495 | 1.463 | 1.527 |  |
|  | Friday | 1.696 | 1.661 | 1.732 |  |

**Table S8.** Adjusted relative risks of respiratory hospital admissions associated with the 2009 dust storm. Excludes reporting of smoothing cubic splines terms used for time.

| **Variable** | **Category** | **RR** | **95% LCI** | **95% UCI** | **p-value** |
| --- | --- | --- | --- | --- | --- |
| 23/9/09-6/10/09 | | 0.898 | 0.853 | 0.945 | <.001 |
| Temperature | Lag 1 | 0.987 | 0.976 | 0.998 | 0.019 |
|  | Lag 2 | 1.001 | 0.987 | 1.015 | 0.926 |
|  | Lag 3 | 0.983 | 0.970 | 0.995 | 0.007 |
|  | Lag 4 | 0.987 | 0.959 | 1.015 | 0.361 |
|  | Lag 5 | 0.985 | 0.962 | 1.007 | 0.180 |
| Humidity | Lag 1 | 1.022 | 1.007 | 1.038 | 0.004 |
|  | Lag 2 | 1.004 | 0.984 | 1.023 | 0.723 |
|  | Lag 3 | 1.026 | 1.008 | 1.046 | 0.006 |
|  | Lag 4 | 1.014 | 0.995 | 1.033 | 0.145 |
|  | Lag 5 | 1.017 | 1.002 | 1.032 | 0.026 |
|  | Lag 6 | 1.046 | 1.004 | 1.090 | 0.030 |
|  | Lag 7 | 0.992 | 0.979 | 1.006 | 0.257 |
| SO2 | Lag 1 | 0.997 | 0.984 | 1.010 | <.001 |
|  | Lag 2 | 0.976 | 0.958 | 0.993 | <.001 |
|  | Lag 3 | 0.988 | 0.974 | 1.003 | 0.120 |
|  | Lag 4 | 0.981 | 0.965 | 0.996 | <.001 |
|  | Lag 5 | 0.988 | 0.972 | 1.004 | 0.130 |
|  | Lag 6 | 0.974 | 0.944 | 1.004 | <.001 |
|  | Lag 7 | 1.007 | 0.989 | 1.025 | <.001 |
| O3 | Lag 1 | 1.007 | 0.979 | 1.035 | 0.640 |
|  | Lag 2 | 1.018 | 0.985 | 1.052 | 0.282 |
|  | Lag 3 | 1.012 | 0.981 | 1.043 | 0.455 |
|  | Lag 4 | 1.025 | 1.005 | 1.045 | 0.015 |
|  | Lag 5 | 1.028 | 0.959 | 1.101 | 0.436 |
|  | Lag 6 | 1.000 | 0.969 | 1.032 | 0.997 |
| NO2 | Lag 1 | 0.975 | 0.944 | 1.007 | 0.118 |
|  | Lag 2 | 0.967 | 0.933 | 1.002 | 0.063 |
|  | Lag 3 | 0.983 | 0.951 | 1.016 | 0.314 |
|  | Lag 4 | 0.969 | 0.935 | 1.005 | 0.089 |
|  | Lag 5 | 0.997 | 0.974 | 1.020 | 0.785 |
|  | Lag 6 | 0.952 | 0.884 | 1.025 | 0.194 |
|  | Lag 7 | 0.952 | 0.915 | 0.991 | 0.016 |
| CO | Lag 1 | 1.023 | 1.007 | 1.038 | 0.004 |
|  | Lag 2 | 1.015 | 0.993 | 1.037 | 0.179 |
|  | Lag 3 | 1.021 | 1.004 | 1.039 | 0.018 |
|  | Lag 4 | 1.030 | 1.010 | 1.050 | 0.003 |
|  | Lag 5 | 1.030 | 0.988 | 1.073 | 0.164 |
|  | Lag 6 | 1.005 | 0.981 | 1.030 | 0.672 |
| Day of week | Sunday | 0.691 | 0.675 | 0.708 | <.001 |
|  | Monday | 1.575 | 1.545 | 1.606 |  |
|  | Tuesday | 1.768 | 1.735 | 1.802 |  |
|  | Wednesday | 1.742 | 1.709 | 1.775 |  |
|  | Thursday | 1.727 | 1.695 | 1.760 |  |
|  | Friday | 1.925 | 1.890 | 1.961 |  |

**Table S9.** Adjusted relative risks of asthma hospital admissions associated with the 2009 dust storm. Excludes reporting of smoothing cubic splines terms used for time.

| **Variable** | **Category** | **RR** | **95% LCI** | **95% UCI** | **p-value** |
| --- | --- | --- | --- | --- | --- |
| 23/9/09-6/10/09 | | 1.141 | 0.991 | 1.313 | 0.066 |
| Temperature | Lag 1 | 0.986 | 0.947 | 1.026 | 0.478 |
|  | Lag 2 | 1.077 | 1.014 | 1.143 | 0.015 |
|  | Lag 3 | 1.011 | 0.961 | 1.065 | 0.672 |
|  | Lag 4 | 1.043 | 0.989 | 1.100 | 0.119 |
|  | Lag 5 | 0.991 | 0.934 | 1.051 | 0.762 |
|  | Lag 6 | 1.081 | 0.966 | 1.209 | 0.177 |
|  | Lag 7 | 0.932 | 0.860 | 1.011 | 0.089 |
| Humidity | Lag 1 | 0.953 | 0.912 | 0.996 | 0.034 |
|  | Lag 2 | 0.922 | 0.875 | 0.972 | 0.003 |
|  | Lag 3 | 0.974 | 0.938 | 1.012 | 0.179 |
|  | Lag 4 | 0.893 | 0.799 | 0.999 | 0.047 |
|  | Lag 5 | 1.006 | 0.968 | 1.045 | 0.768 |
| SO2 | Lag 1 | 0.974 | 0.933 | 1.016 | 0.223 |
|  | Lag 2 | 1.044 | 0.984 | 1.108 | 0.151 |
|  | Lag 3 | 1.009 | 0.962 | 1.058 | 0.706 |
|  | Lag 4 | 1.073 | 1.019 | 1.129 | 0.007 |
|  | Lag 5 | 1.004 | 0.954 | 1.057 | 0.869 |
|  | Lag 6 | 1.108 | 1.002 | 1.224 | 0.045 |
|  | Lag 7 | 1.032 | 0.972 | 1.095 | 0.298 |
| O3 | Lag 1 | 0.920 | 0.858 | 0.987 | 0.020 |
|  | Lag 2 | 0.888 | 0.840 | 0.940 | <.001 |
|  | Lag 3 | 0.849 | 0.724 | 0.994 | 0.042 |
|  | Lag 4 | 1.109 | 1.017 | 1.209 | 0.019 |
| NO2 | Lag 1 | 0.963 | 0.925 | 1.003 | 0.070 |
|  | Lag 2 | 0.866 | 0.748 | 1.001 | 0.052 |
|  | Lag 3 | 0.881 | 0.814 | 0.953 | 0.002 |
| CO | Lag 1 | 1.016 | 0.966 | 1.068 | 0.545 |
|  | Lag 2 | 1.114 | 1.038 | 1.195 | 0.003 |
|  | Lag 3 | 1.063 | 1.003 | 1.126 | 0.039 |
|  | Lag 4 | 1.116 | 1.054 | 1.182 | <.001 |
|  | Lag 5 | 1.061 | 0.925 | 1.216 | 0.399 |
|  | Lag 6 | 0.927 | 0.856 | 1.003 | 0.061 |
| Day of week | Sunday | 1.008 | 0.945 | 1.075 | <.001 |
|  | Monday | 1.441 | 1.358 | 1.530 |  |
|  | Tuesday | 1.380 | 1.300 | 1.466 |  |
|  | Wednesday | 1.329 | 1.251 | 1.412 |  |
|  | Thursday | 1.278 | 1.202 | 1.359 |  |
|  | Friday | 1.375 | 1.294 | 1.460 |  |
